# Supplementary material for: Discovery of Screening Biomarkers for Major Depressive Disorder in Remission by Proteomic Approach
Source: Diagnostics (Basel). 2021 Mar 17;11(3):539. doi: 10.3390/diagnostics11030539 (PMC8002827; doi:10.3390/diagnostics11030539)
Supplement: Supplementary file 1 [file diagnostics-11-00539-s001.pdf]

Supplementary Materials

a

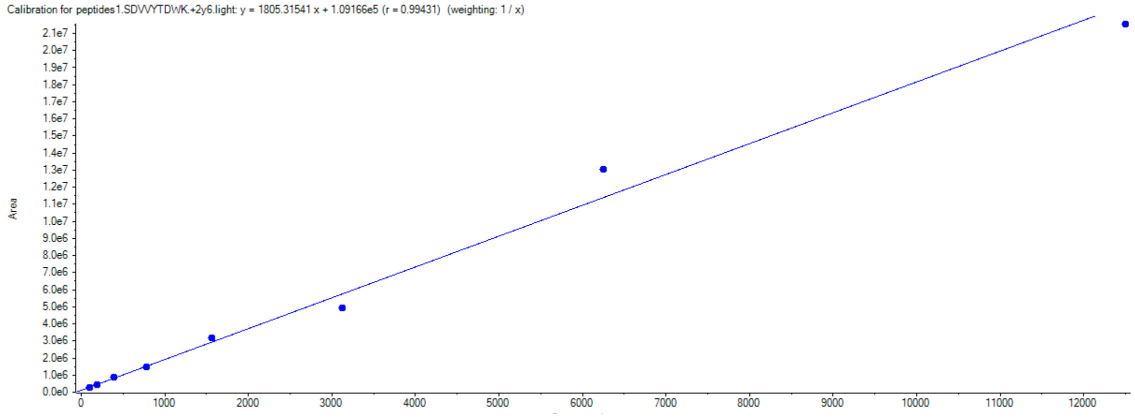

b

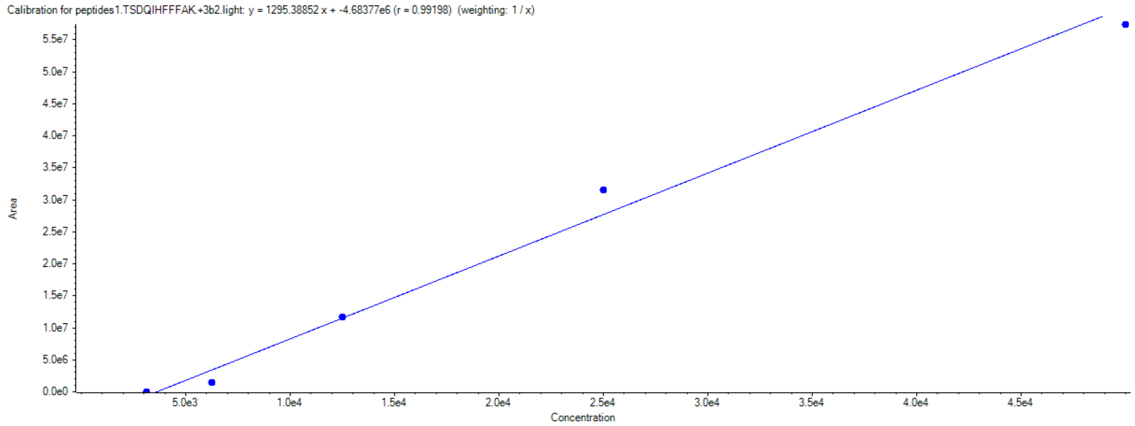

c

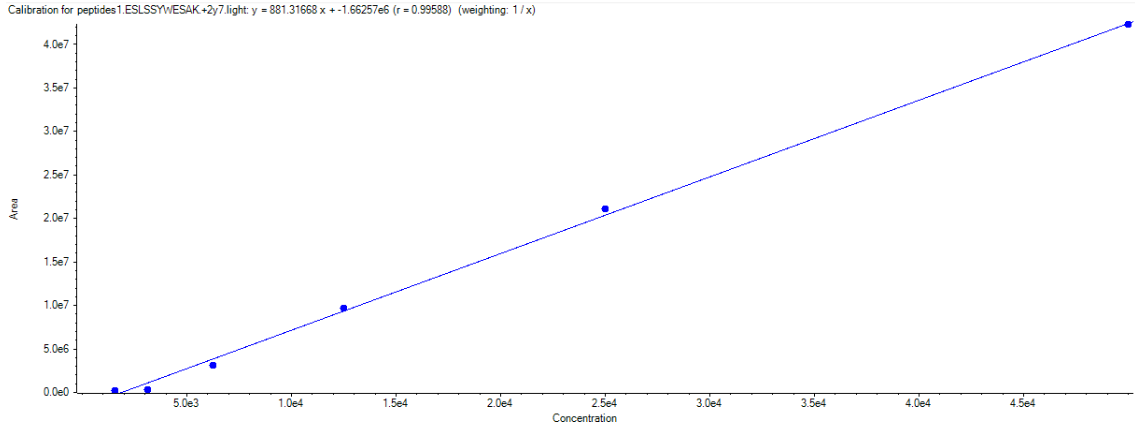

d

Calibration for peptides1.SLPVSDSVLSGFEQR.+2y7.CE_35.0.light: y = 837.68756 x + -2.91287e5 (r = 0.99007) (weighting: 1 / x)

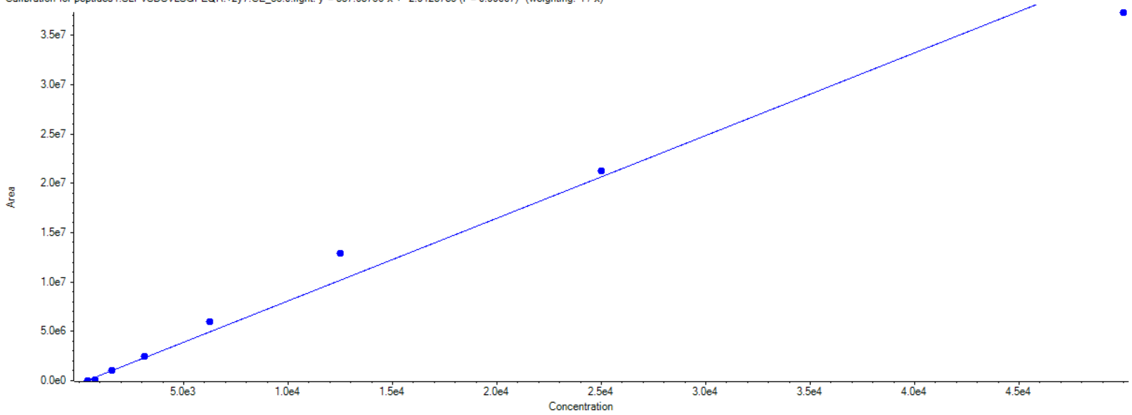

e

Calibration for peptides1.SLEYDLSFNQIAR.+2y8.light: y = 489.13481 x + -3.00242e5 (r = 0.99344) (weighting: 1 / x)

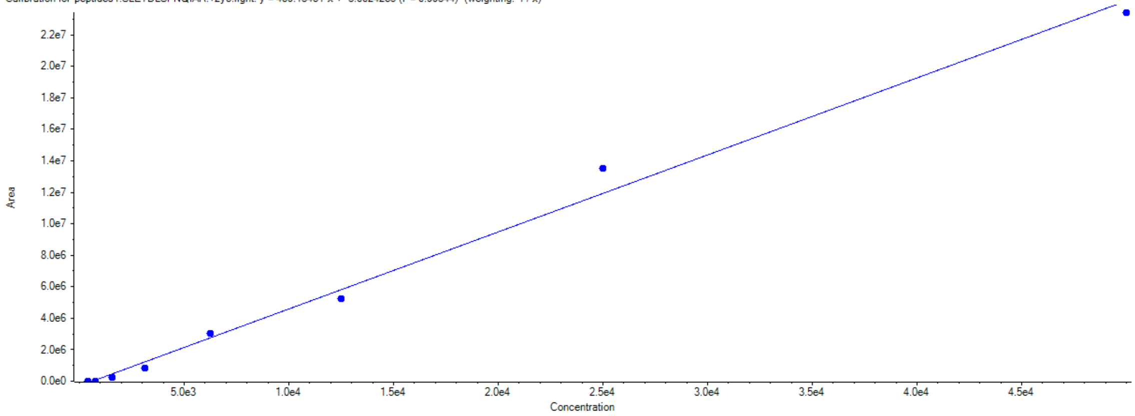

f

Calibration for peptides1.ELLESYIDGR.+2y7.light: y = 1210.48650 x + -1.82496e5 (r = 0.99369) (weighting: 1 / x)

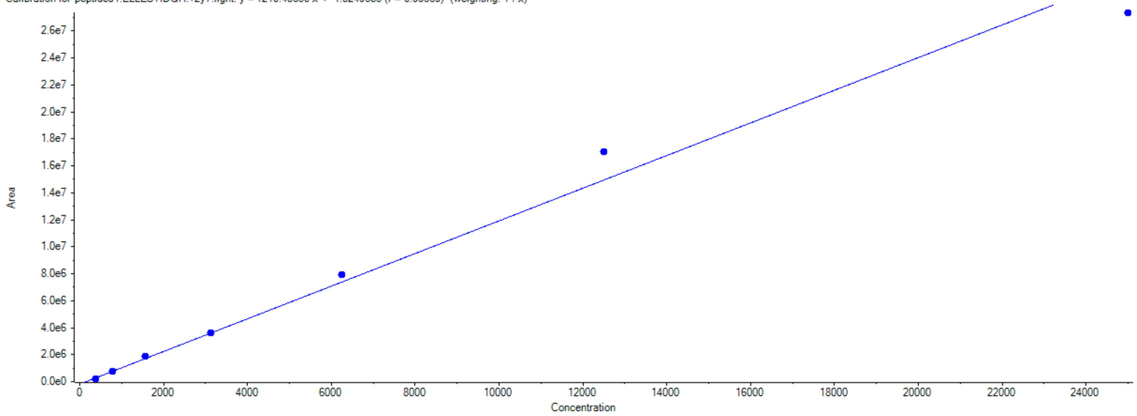

g

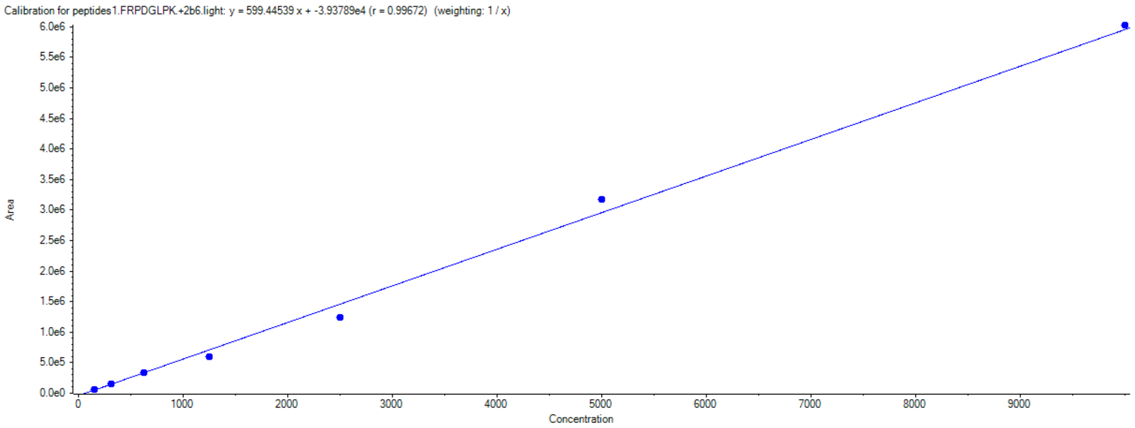

Figure S1. Peptide calibration curves for MRM analysis. (a) Calibration curve of alpha-1-acid glycoprotein 1 (SDVVYTDWK, $r^2 = 0.99431$). (b) Calibration curve of antithrombin-III (TSDQIHFFFAK, $r^2 = 0.99198$). (c) Calibration curve of apolipoprotein C-II (ESLSSYWESAK, $r^2 = 0.99588$). (d) Calibration curve of complement component C8 gamma chain (SLPVSDSVLSGFEQR, $r^2 = 0.99007$). (e) Calibration curve of lumican (SLEYDLSFNQIAR, $r^2 = 0.99344$). (f) Calibration curve of prothrombin (ELLESYIDGR, $r^2 = 0.99369$). (g) Calibration curve of serum amyloid A-4 protein (FRPDGLPK, $r^2 = 0.99672$).
